# Regionally Specific Regulation of Sensorimotor Network Connectivity Following Tactile Improvement

**DOI:** 10.1155/2017/5270532

**Published:** 2017-11-02

**Authors:** Stefanie Heba, Melanie Lenz, Tobias Kalisch, Oliver Höffken, Lauren M. Schweizer, Benjamin Glaubitz, Nicolaas A. J. Puts, Martin Tegenthoff, Hubert R. Dinse, Tobias Schmidt-Wilcke

**Affiliations:** ^1^Department of Neurology, BG University Hospital Bergmannsheil, Bürkle-de-la-Camp-Platz 1, 44789 Bochum, Germany; ^2^Institute for Neuroinformatics, Neural Plasticity Lab, Ruhr-University Bochum, Universitätsstr. 150, 44801 Bochum, Germany; ^3^Russell H. Morgan Department of Radiology and Radiological Science, The Johns Hopkins University School of Medicine, 601 North Caroline Street, Baltimore, MD 21287-0006, USA; ^4^F.M. Kirby Research Center for Functional Brain Imaging, Kennedy Krieger Institute, 707 North Broadway, Baltimore, MD 21205, USA; ^5^Department of Neurology, St. Mauritius Therapieklinik, Lehrkrankenhaus der Universität Düsseldorf, 40670 Meerbusch, Germany; ^6^Institute of Clinical Neuroscience and Medical Psychology, University of Düsseldorf, 40225 Düsseldorf, Germany

## Abstract

Correlations between inherent, task-free low-frequency fluctuations in the blood oxygenation level-dependent (BOLD) signals of the brain provide a potent tool to delineate its functional architecture in terms of intrinsic functional connectivity (iFC). Still, it remains unclear how iFC is modulated during learning. We employed whole-brain resting-state magnetic resonance imaging prior to and after training-independent repetitive sensory stimulation (rSS), which is known to induce somatosensory cortical reorganization. We investigated which areas in the sensorimotor network are susceptible to neural plasticity (i.e., where changes in functional connectivity occurred) and where iFC might be indicative of enhanced tactile performance. We hypothesized iFC to increase in those brain regions primarily receiving the afferent tactile input. Strengthened intrinsic connectivity within the sensorimotor network after rSS was found not only in the postcentral gyrus contralateral to the stimulated hand, but also in associative brain regions, where iFC correlated positively with tactile performance or learning. We also observed that rSS led to attenuation of the network at higher cortical levels, which possibly promotes facilitation of tactile discrimination. We found that resting-state BOLD fluctuations are linked to behavioral performance and sensory learning, indicating that network fluctuations at rest are predictive of behavioral changes and neuroplasticity.

## 1. Introduction

The acquisition of new skills, or the recovery of function after damage to the central nervous system, requires changes in neuronal connections. The cellular mechanisms of this key feature of neuronal networks can be well characterized by means of electrophysiology in animal models [1–4], whereas the investigation of cortical plasticity in humans is typically based on observed changes in task-evoked responses [5–8]. Temporal coherence in neuronal discharges distributed over a wide region is the physiological underpinning of resting-state networks (RSN) [9, 10]. On a whole-brain scale, correlations between inherent, so-called task-free low-frequency fluctuations in blood oxygenation level-dependent (BOLD) signals of the brain provide a potent tool to delineate the functional architecture of the brain [11–15]. RSN have been in the focus of scientific research ever since Biswal et al. [16] showed the spatial congruency between regions activated during task-related functional magnetic resonance imaging (fMRI) and regions with highly correlated low-frequency BOLD time courses during resting-state fMRI (rs-fMRI), giving rise to the term intrinsic functional connectivity ((i)FC). A logical consequence is to utilize resting-state fMRI to investigate learning-induced plasticity, which is reflected in changes in task-free BOLD fluctuations after training and/or learning. This method has been successfully employed for both healthy controls [11, 17, 18] and patients with stroke or carpal tunnel syndrome [19, 20].

A particularly reliable method to induce plastic changes in the tactile domain is training-independent repetitive sensory stimulation (rSS), a form of attention-independent sensory learning [21, 22]. RSS consisting of high-frequency intermittent tactile stimulation can be regarded as a long-term potentiation- (LTP-) like protocol that enforces Hebbian learning [23] which provides a biological basis for erroneous learning methods for education and memory rehabilitation, thereby linking cellular plasticity mechanisms to human perceptual learning [5, 24, 25]. Several studies have shown that rSS applied to the index finger improves tactile spatial 2-point discrimination (2ptD) of that finger, presumably via functional reorganization within the somatosensory cortex [5, 26]. Haag et al. [27] were the first to link local connectivity in terms of regional homogeneity to tactile performance at baseline. However, it is as yet unknown whether, and in which way, rSS modulates iFC. Prior studies using electroencephalography (EEG) following rSS revealed significant changes in the mu-rhythm coherency in the sensorimotor (SEMO) system [28]. Still, it remains challenging to identify the distinct cortical areas where changes in functional coupling lead to enhanced effectiveness of neuronal information transfer.

We therefore employed whole-brain resting-state fMRI prior to and after rSS to investigate which areas in the SEMO network are susceptible to training-independent tactile plasticity using human subjects. We hypothesized that intrinsic FC in somatosensory areas (iFC_SEMO_) would increase in those brain regions that primarily receive afferent input. Also, we expected a correlation between iFC modulations and behavioral measures such as 2ptD thresholds and gain in 2ptD due to rSS.

## 2. Materials and Methods

### 2.1. Subjects

Twenty-four subjects (all right-handed) with no previous history of psychological disorders or any known hand or head injuries were enrolled in the study. Six subjects were excluded from further analysis due to either excessive movement in the scanner, incomplete data, or use of medication. This resulted in 18 subjects (10 men, 8 women; aged 23.8 ± 3.5 years), all of whom fell within the normal range of depression and trait anxiety levels, as assessed using the Beck Depression Inventory (BDI, [29]) and State-Trait Anxiety Inventory (STAI, [30]), respectively. Subjects gave their written informed consent and received monetary compensation at the end of the protocol. The experimental protocol had been approved by the local ethics committee of the Ruhr-University Bochum and was performed in accordance with the Declaration of Helsinki. The two-point discrimination thresholds that were collected on this group of subjects have been published previously [27, 31].

### 2.2. Assessment of 2-Point Discrimination Thresholds

2ptD thresholds were assessed on the tip of the index finger (D2) of both hands by using the method of constant stimuli [5, 25, 26, 32–34].

**Figure 1:**
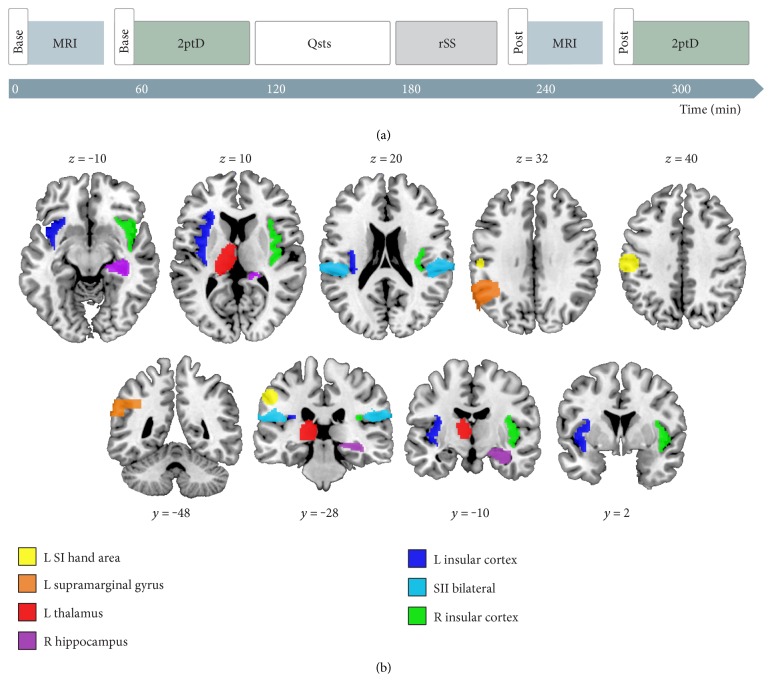
Experimental schedule and anatomical ROI for small volume correction. (a) For each subject, we assessed resting state MRI, followed by the assessment of 2ptD thresholds of the index fingers of both the left and right hands. After baseline assessments, subjects filled out questionnaires concerning personality traits to ensure that all subjects fell within the normal range of depression and trait anxiety levels (Qsts). During the subsequent rSS intervention, one hand received 45 min of intermittent high-frequency stimulation, whereas the other hand served as a control condition. The time between the end of rSS and the start of the second RS session ranged between 35 and 70 min (50.0 ± 1.98 min). (b) Representative slices of anatomical a priori (i.e., previously deduced) regions-of-interest used for the small volume correction of statistical results. We focused on brain regions known to participate in the processing of somatosensory stimulation.

2ptD thresholds were assessed at a fixed location on the skin of the fingertips by rapidly switching between stimuli. In short, stimuli consisted of 7 pairs of brass needles with individual spacing ranging from 0.7 to 2.5 mm in increments of 0.3 mm and a single needle as zero distance (control condition). The subjects were instructed to place their finger on the support and to maintain this initial position of the finger throughout the experiment. Probes were presented 8 times in a randomized order resulting in 64 trials per session. Subjects were not informed about the ratio of paired to single needles being 7 : 1. The participants had to decide immediately after stimulus contact if they had the sensation of 1 or 2 needles being applied by reporting the percept of a single needle, or any ambiguous stimulus, as “1” and the distinct percept of 2 needle tips as “2.” The tip spacing was plotted against the percentage of double-tip responses given and fitted by a binary logistic regression, resulting in a psychometric function where chance level of the sigmoid fit marked the individual 2ptD threshold. The behavioral gain was calculated according to the following equation:

(1) $$\begin{array}{c} \left(\frac{base-post}{base}\right)\times 100, \end{array}$$

with a positive gain indicating an improvement in tactile perception, that is, lower 2ptD thresholds after rSS. All subjects underwent one training session in order to familiarize themselves with the testing procedure (Test). A second session prior to rSS served as a baseline (Base). A third assessment was performed 45–90 min after rSS intervention (Post). Changes of interest were between the Base and the Post sessions (Figure 1(a)).

To provide evidence that a change in discrimination sensitivity was not due to a change in response criterion, we calculated the discrimination index d-prime (d′). The d′ value equals the difference between the *z*-transform of the hit rate (the probability of discriminating 2 tips whenever 2 tips are presented, *z*(H)) and the *z*-transform of the false alarm rate (the probability of detecting 2 tips when only 1 is present, *z*(F)) with d′ = *z*(H) − *z*(F). To carry out the numerical calculation in case of zero false alarm rates, the false alarm rate was set to 0.0165 (1/2N, with *N* = 8 being the number of control trials). Effect size was calculated as Cohen's *d* [35].

### 2.3. Electrical Repetitive Sensory Stimulation Protocol

rSS was applied for 45 min to the dominant right hand. The rSS sequence was applied to the fingertips of all digits and consisted of stimulus trains of 2 s (including 2 × 0.5 s ramps, single-pulse duration: 0.2 ms (square), frequency: 20 Hz) and intertrain intervals of 5 s, played back from a digital storage that triggered a standard TENS device (Pierenkemper, Germany). Electrical pulses were transmitted by adhesive surface electrodes (4 cm^2^, Pierenkemper) fixed to the first and third segment of each finger (cathode placed proximal) and current intensity was adjusted individually for each subject (mean intensity 9.48 ± 3.37 mA) to maintain a stable percept of stimulating the fingertips across participants.

### 2.4. MR Protocols

Participants were scanned on a Philips 3.0T Achieva X scanner using a 32-channel head coil. High-resolution, T1-weighted, structural images (MPRAGE, TR/TE: 8.5/3.9 ms, voxel (vx) size (1 mm)^3^ isotropic) were acquired to enable tissue segmentation.

For acquisition of functional images (Gradient-echo EPI, TR/TE: 2500/35 ms, flip angle: 90 deg, field of view (FOV): 224 × 232 mm, 39 axial slices, slice thickness: 3 mm, no gap, 200 scans, no dummy scans, total acquisition time: 8 min 37 s), participants were instructed to close their eyes and “not to think about anything in particular.” For image converting, the first 10 images during which the BOLD signal reaches steady state were discarded from further analysis to remove nonsteady-state effects caused by T1 saturation.

### 2.5. Image Preprocessing and Independent Component Analysis

Preprocessing of resting-state functional images was performed with the preprocessing routine provided by the functional connectivity toolbox CONN (version 14.n; [36]) and included slice time correction, spatial realignment and unwarping, normalization to the SPM8 MNI template, interpolation to (2 mm)^3^ isotropic voxel, and smoothing with an isotropic 6 mm Gaussian kernel. Images were centered to mean; no filtering and no denoising were applied. The acceptable limit for head motion was 2 mm for translational movements and 0.5 deg for rotational movements.

For the independent component analysis (ICA) using the GIFT toolbox (version 3.0a; http://icatb.sourceforge.net/groupica.htm, last accessed 06/09/2017), data dimensionality was reduced by two principal component analysis (PCA) steps to 51 on subject level and after concatenation of subjects and sessions to 34 which is the estimated number of components using minimum description length (MDL) criteria. InfoMax group ICA was performed to decompose the data into 34 independent components. ICA was repeated 20 times using ICASSO [37], starting each time from a random initial point. Reliability of decomposition was validated by ICASSO results showing compact clusters. Subject-specific spatial maps (SM) and time courses (TC) of independent components were reconstructed using the GICA3 back-reconstruction method. Reconstructed SM of single components were converted to *z*-scores and visually inspected to select the IC most related to the sensorimotor network.

### 2.6. Statistical Analysis

All results are quoted as mean ± SEM, unless stated otherwise. Statistical tests of behavioral data comprised paired *t*-tests and repeated-measures analysis of variance (ANOVA) with factors site and time; for behavioral correlations, Pearson's correlation coefficients are reported (Statistics toolbox and in-house scripts; MATLAB, R2009a, The MathWorks Inc., USA). Subject- and session-specific spatial maps of SEMO-related voxel-wise iFC were fed into a flexible factorial SPM8 design with factors subject (18 levels) and session (2 levels; Base and Post rSS) including age as nuisance variables centered on the overall mean with no interaction. Since the expected effects were rather small, parametrical maps of the contrasts Base > Post and Post > Base were thresholded on voxel level with *P*_unc_ < 0.001 and a minimum cluster size of 10 contiguous voxels. To test the specificity of these clusters, we performed a volume-of-interest (VOI) analysis, that is, the presumed location of the left primary somatosensory cortex (SI) hand area according to the coordinates by Geyer et al. [38] (10 mm sphere at MNI-coordinates *x*/*y*/*z* = −55/−22/41 comprising part of BA 1/2/3/4), as well as region-of-interest (ROI) analyses using six ROIs of (1) the left and right secondary somatosensory cortex (SII), (2) the left thalamus, (3) the left supramarginal gyrus, (4) the left insula cortex, (5) the right insula cortex, and (6) the right hippocampus (WFU PickAtlas version 1.2; [39–41]). Clusters were deemed significant at *P* < 0.05 (after FWE correction for multiple comparisons within the specific ROI).

Group ICA [42] enables the back reconstruction of distinct network components, here the SEMO network, derived from pooled resting-state data to individual subject's whole-brain maps. In contrast to any seed-based approaches, with ICA, we benefit from data-driven separation of whole-brain networks without any confounding signals in a ROI caused by other RSNs of no interest [12]. Whole-brain regression analyses were performed on (a) single session IC19 SMs with 2ptD thresholds as predictor, and (b) calculated difference images (IC19 Post-IC19 Base session) and 2ptD gain as predictor.

## 3. Results

### 3.1. Effects of Repetitive Sensory Stimulation on Tactile Perception

As we reported in our previous study [32], after 45 min of intermittent high-frequency stimulation of all fingers of the right hand, tactile discrimination performance of the stimulated index finger—in terms of 2ptD thresholds—improved by 12% on average (Base: 1.59 ± 0.03 mm, Post: 1.38 ± 0.05 mm; *d*_Cohen_: 5.093), while performance of the nonstimulated left hand index finger (serving as control) remained unaltered, confirming previously reported selective improvements after rSS [34] (repeated-measures ANOVA with factor site *F*_(1, 17)_ = 0.46, *P* = 0.506, factor time *F*_(1, 17)_ = 5.30, *P* = 0.034, and interaction site × time *F*_(1, 17)_ = 18.51, *P* < 0.001). A two-factor repeated-measures ANOVA showed a significant stimulation site × time interaction on tactile sensitivity (*F*_(1, 17)_ = 28.39, *P* < 0.001), such that d′ of the stimulated hand increased significantly more than on the nonstimulated hand [31].

### 3.2. Effects of Repetitive Sensory Stimulation on Sensorimotor Network Connectivity

**Figure 2:**
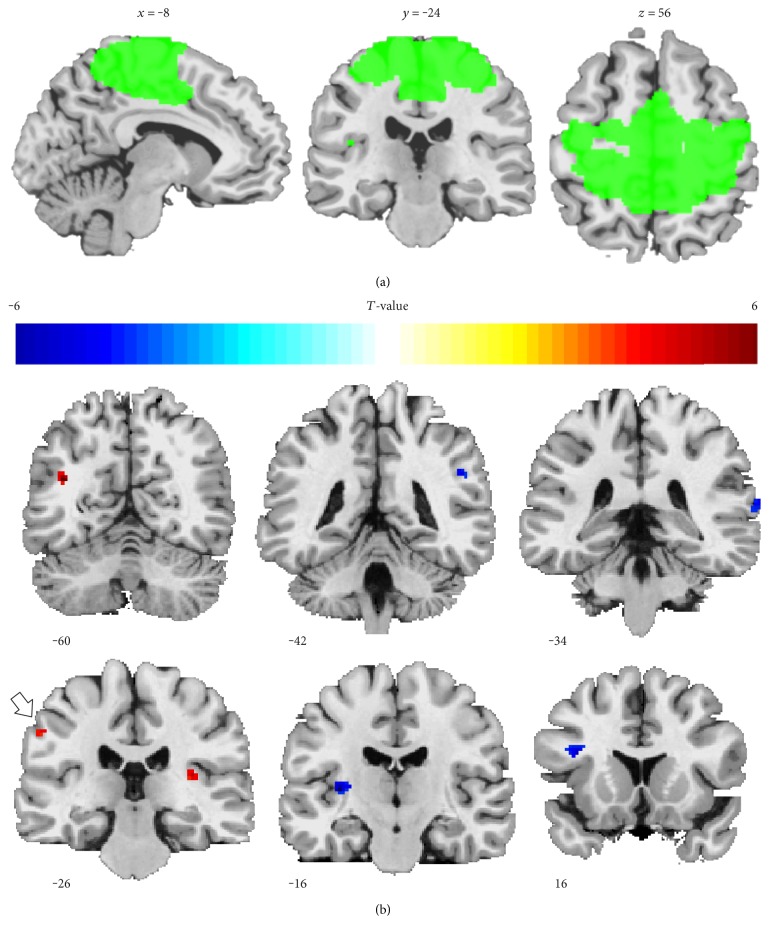
Sensorimotor network component and longitudinal changes in intrinsic network connectivity. (a) Representative sagittal, coronal, and axial slices of the ICA-extracted sensorimotor resting-state pattern on a brain template. The pattern (green) was estimated from a group of 18 subjects. The resulting binary image shows *z*-scaled network connectivity thresholded at *z* > 1 in neurological convention, with coordinates referring to *x*, *y*, and *z* in mm MNI space. (b) GLM analysis of changing iFC_SEMO_ after rSS at *P*_unc._ < 0.001 (*k* > 10 vx). Regions of higher iFC are plotted in red, whereas regions of lower iFC are plotted in blue. An arrow marks the cluster at the left postcentral gyrus (BA 3) which survived a small volume correction (10 mm sphere at *x*/*y*/*z* = −55/−22/41; *P*_FWE_ = 0.016). Coordinates refer to *x*, *y*, and *z* in mm MNI space and neurological convention.

We found substantial overlap between the spatial map of the independent component “IC19” at a *z*-score threshold > 1 and brain areas activated in sensorimotor tasks such as pre- and postcentral gyri, extending from the medial bank of the intrahemispheric fissure close to the superior wall of the Sylvian fissure, including supplementary and premotor areas (Figure 2(a)). As a result, we identified IC19 to represent the sensorimotor network and made it subject to our analyses. Random-effect analyses (*P*_unc._ < 0.001, *k* = 10 vx) of Base-rSS and Post-rSS sessions showed rSS-related rise in iFC_SEMO_ to the left angular gyrus, the right insula cortex, the left postcentral gyrus, the left inferior parietal lobe, the anterior cingulate cortex, and the paracentral lobe. An rSS-related decline of iFC_SEMO_ was observed in the left insula cortex, the left inferior frontal gyrus, the right superior temporal gyrus, the right supramarginal gyrus, and the precuneus (Table 1, Figure 2(b)). Only the clusters in the left postcentral gyrus and the left insula cortex survived corrections for multiple comparisons within the corresponding a priori defined ROIs.

### 3.3. Association between Tactile Discrimination and Functional Connectivity

We performed whole-brain regression analyses to estimate the magnitude of correlation between iFC_SEMO_ and 2ptD thresholds, as well as between changes in iFC_SEMO_ and 2ptD gain.

**Figure 3:**
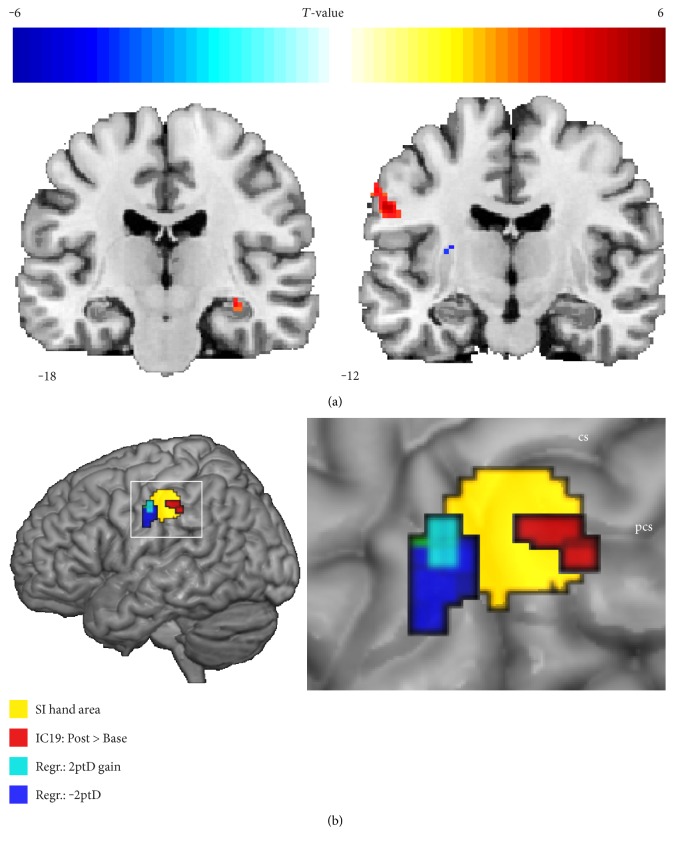
2ptD performance linking to iFC_SEMO_. (a) Representative coronal slices of statistical maps derived from regression analyses showing changes in whole-brain iFC_SEMO_ with 2ptD gain as predictor (left), and whole-brain iFC_SEMO_ after rSS with 2ptD thresholds after rSS as predictor (right). Warm colors on the left-hand slice correspond to a positive correlation between iFC and 2ptD gain, whereas warm colors on the right-hand slice relate to negative correlations between iFC and 2ptD thresholds representing improved discrimination performance. Coordinates refer to *y* in mm MNI space and neurological convention, and individual clusters are reported at whole brain *P*_unc._ < 0.001 (*k* > 10 vx). (b) Whole-brain 3D-render depicting clusters in the vicinity of the SI hand area ROI (yellow). Red voxel refer to increased iFC_SEMO_ after rSS. Resulting voxel of a regression between iFC_SEMO_ and 2ptD gain are given in cyan, whereas resulting voxel of a regression between iFC_SEMO_ and 2ptD thresholds are colored in dark blue. Cluster are reported at *P*_unc._ < 0.001 (*k* > 10 vx). Inset at right displays enlarged view of marked region. cs: central sulcus; pcs: postcentral sulcus.

We found no significant correlations between baseline 2ptD thresholds and baseline iFC_SEMO_. Following rSS application, we found a significant correlation between 2ptD thresholds and iFC_SEMO_ within the postcentral gyrus receiving input from the stimulated hand, that is, the lower the postinterventional thresholds, the higher the postinterventional iFC_SEMO_ in the hand area of the postcentral gyrus (Figure 3(a), Table 2). This result was significant when corrected for multiple comparisons within the hand area VOI (*x*/*y*/*z* = −58/−12/30; *P*_FWE_ = 0.039).

Further associations (at an uncorrected threshold) between behavioral gain and changes in iFC_SEMO_ were found in parts of the right supramarginal gyrus and left cerebellar lobule VI, as well as in the right middle occipital gyrus, and the hand area of the left postcentral gyrus (all *P*_unc._ < 0.001, *k* > 10 vx; Table 2). The only significant correlation between behavioral gain and changes in iFC_SEMO_ was found in the right hippocampus (*x*/*y*/*z* = 32/−18/12; *P*_FWE_ = 0.040). In this region, an improvement in tactile discrimination was associated with strengthened iFC_SEMO_.  Figure 3(b) depicts those regions correlating with either 2ptD thresholds (at a corrected threshold of *P*_FWE_ < 0.05) or 2ptD gain (at an uncorrected threshold of *P*_unc_ < 0.001), which are in close proximity to the cluster of rSS-related increase in iFC_SEMO_.

## 4. Discussion

In this study, we investigated changes in the iFC of the sensorimotor RSN induced by intermittent high-frequency tactile stimulation. With respect to timing, this stimulation resembles an LTP-like protocol used in synaptic plasticity research [21].

To explore the role of iFC in human tactile learning, we acquired resting-state fMRI as well as 2ptD thresholds before and after rSS and investigated the sensorimotor resting-state network by means of a group ICA.

### 4.1. RSS Causes Multi-Level Changes of the Sensorimotor Resting-State Network

Many lines of evidence suggest that the effects of rSS are based on changes in synaptic efficiency and transmission [25, 32–34, 43]. Besides, neuronal activity in terms of the local field potential (LFP) and electrophysiological measures such as power band activity are the foundation of changes in BOLD signal strength [9]. Task-based fMRI and resting-state EEG data showed that rSS can induce cortical reorganization in SI and SII [2, 5, 26, 28]. Here, we extend these findings by using resting-state fMRI data to explore the functional connectivity of the sensorimotor network after rSS-induced learning.

Our behavioral data confirmed a significant improvement in tactile discrimination of the stimulated hand after rSS, and resting-state fMRI data reveals changes in the iFC_SEMO_ at the corresponding hand representation in SI.

Since rSS is not reliant on attentional focus during stimulation, we had strong a priori hypotheses for brain regions comprising traditional low-level somatosensory areas: temporal coherence in neuronal discharge is known to emerge, for example, during frequency discrimination learning in the SI of owl monkeys, and is assumed to account for the reported improvement in behavior [44]. However, we did not exclude the involvement of high-level hubs engaged in cognitive functions such as attention and memory [6]. Indeed, we also found stronger iFC_SEMO_ following rSS in the left angular gyrus and the right insula—regions known to play a role in finger gnosia [45]. Notably, we also observed that the prolonged afferent input applied during rSS led to attenuation of the SEMO network at higher cortical levels, such as the temporoparietal junction (TPJ), midposterior insula, and Rolandic operculum. Previous studies showed that especially the right TPJ is activated by unattended stimuli with task-relevant features requiring attention, leading to stimulus directed reorientation [46]. Therefore, the right TPJ acts as an interrupt to ongoing processes and is associated with the filtering of sensory events to optimize behavioral performance [47, 48]. Based on our current findings, we conclude that the deterioration of iFC_SEMO_ at the right TPJ promotes the ability to maintain set for sensory input and discrimination.

### 4.2. Tactile Learning Is Linked to Brain Network Connectivity at Rest

In addition to rSS-related changes in iFC_SEMO_, we were also interested in identifying regions whose network strength was related to tactile perception and/or tactile learning. We found a significant correlation between iFC_SEMO_ and 2ptD thresholds after rSS in regions adjacent to the area that displayed an intervention-driven increase in iFC_SEMO_.

RSS-induced Hebbian mechanisms are known to increase synaptic activity and/or efficiency. Additionally, the driving input might recruit (intra-)cortical connections, propagating the neuronal signal horizontally, thereby providing a potential explanation for the enlargement of cortical maps observed after stimulation [5, 24, 25]. Only during synchronous input, higher temporal coherence of neuronal discharge between single digit representations has been found, as demonstrated by seed-based approaches [49]. Our present results from task-free rs-fMRI show that this increased neural synchronization within the SEMO network still persists after stimulation has ceased and is strongly correlated with both the improvement and performance levels of tactile discrimination after stimulation. These episodic changes in coherence may support the early onset of (structural) changes in microcircuitry (see [50] for review). We assume that the neuronal oscillation might be the functional surrogate of behavioral improvement.

### 4.3. Higher Level Brain Regions Exhibit Changes in the Sensorimotor Network

Importantly, our data support an association between tactile learning induced by rSS and the iFC_SEMO_ of higher-order associative brain regions (cerebellum and hippocampus). Even though some of the clusters did not reach statistical significance, or were not in the focus of our a priori hypotheses, their spatial distribution and direction of effect provides interesting insights. For example, somatomotor representations are known to exist in contralateral cerebellar lobules V and VI [51, 52], comprising afferent and efferent connections with primary motor cortex, respectively. Also, longitudinal enhancement has been reported for the FC between SI and contralesional cerebellar lobules I–VI during stroke recovery [19], which, moreover, was associated with regain of function in a touch discrimination task. The hippocampus is considered to be the major hub in memory and learning processes [53]. The significant correlation between behavioral improvement and stronger iFC_SEMO_ in the right hippocampus therefore provides evidence for the integration of attention and memory processes during or following rSS. Although the efficacy of rSS is independent of active attention to stimulation, the incoming stimuli have to be processed and stored as rSS goes unattended, yet not unnoticed. There is electrophysiological evidence from animal studies which proves the participation of the hippocampus in the processing of tactile stimuli. Information on discriminative touch and stimulus identity can be recorded in the CA1 region of vibrissal sensing rats [54], and hippocampal local field potentials are altered during collection of stimulus features to enhance the efficiency of integration of stimulus information and memory and decision-making centers [55]. The same mechanisms may apply for human neural processing as well. However, the possibility cannot be excluded that changes in hippocampal BOLD-related signals at rest might be a mere neurophysiological response to prior task engagement (what we here consider to be the rSS intervention), rather than an effect of memory consolidation.

Considering limitations of our study, it is important to mention the uncorrected statistical thresholds of some of our findings, especially those concerning regions outside the classical somatosensory system. Accordingly, these findings should be viewed with caution; however, we considered them worth reporting as they might set the path for new a priori hypotheses of future studies.

Functional connectivity analyses in general allow no assumptions on causality, or on the directedness of influence. Against this background, it is conceivable that connectivity between two regions/components is driven by a third region not identified in the analysis. More sophisticated approaches to the exploration of effective connectivity and the relationship between functional and structural connectivity are needed to overcome such methodological shortcomings in future studies.

## 5. Conclusions

Our findings provide evidence for effects of rSS on the intrinsic network connectivity in the resting-state sensorimotor system. Local upregulations in iFC are found for brain regions primarily receiving somatosensory input, or regions recruited during stimulus discrimination. Notably, we also observed rSS leading to attenuation of the SEMO network on higher cortical levels. Moreover, resting-state BOLD fluctuations were linked to behavioral performance and sensory learning, thus providing further insight into the importance of network fluctuations at rest.

## Figures and Tables

**Table 1:** Anatomical locations, size, *T*-values and peak coordinates (in MNI space) for brain areas where the strength of connectivity was found to change after rSS in the resting-state whole-brain sensorimotor network at *P*_unc._ < 0.001 (*k* > 10 vx). Voxel are reported as 2 × 2 × 2 mm^3^.

Location	BA	Voxel	*T*-value	*x*	*y*	*z*	*P*_FWE_^∗^
*Pre > post*							
L insular cortex		56	−6.61	−34	−16	6	0.007
L IFG (pars triangularis)		36	−5.54	−38	16	20	—
R superior temporal gyrus	22	18	−4.41	70	−36	14	—
R supramarginal gyrus (IPL)		14	−4.43	46	−40	30	—
R precuneus	7	10	−4.35	18	−62	52	—
*Post > pre*							
L angular gyrus	39	23	6.11	−40	−60	22	—
R insula cortex		20	5.80	34	−28	16	0.199
L postcentral gyrus	3, 2	20	4.51	−56	−26	40	0.014
L inferior parietal lobe		14	4.46	−28	−44	40	—
L anterior cingulate cortex		10	4.39	−6	36	−4	—
R paracentral lobe	4, 6	10	4.26	4	−40	62	—

^∗^Small volume corrected on cluster level. BA: Brodmann area; IFG: inferior frontal gyrus; IPL: inferior parietal lobe; L: left; R: right.

**Table 2:** Anatomical location, size, *T*-value, and peak coordinates (in MNI space) for whole-brain multiple regressions of the resting-state sensorimotor network with 2ptD thresholds or 2ptD gain as covariate of interest. Clusters are reported at whole brain *P*_unc._ < 0.001 (*k* > 10 vx). Voxels are reported as 2 × 2 × 2 mm^3^.

*−TPD_baseline_*								*TPD_baseline_*							
*Location*	*BA*	*Voxel*	*T-value*	*x*	*y*	*z*	*P*_FWE_^∗^	*Location*	*BA*	*Voxel*	*T-value*	*x*	*y*	*z*	*P*_FWE_^∗^
L frontal pole	10	18	4.62	−24	64	16	—	L precentral gyrus	6	18	6.22	−38	−4	48	—
								L angular gyrus	—	35	5.86	−34	−62	40	—
								L calcarine gyrus	17	38	5.52	−6	−100	−4	—
								L parahippocampal gyrus	—	16	5.04	−14	−14	−18	—
								L precuneus	—	10	4.88	−6	−58	66	—
								R temporal pole	38	11	3.39	52	16	−10	—
								R rolandic operculum	22	13	3.37	64	4	6	—

*−TPD_post_*								*TPD_post_*							
*Location*	*BA*	*Voxel*	*T-value*	*x*	*y*	*z*	*P*_FWE_^∗^	*Location*	*BA*	*Voxel*	*T-value*	*x*	*y*	*z*	*P*_FWE_^∗^
L postcentral gyrus	4	69	5.84	−58	−12	30	0.039	L parahippocampal gyrus	—	14	5.85	−30	−30	−16	—
L cerebellum (VI)	—	16	5.13	−32	−44	−28	—	L inferior temporal gyrus	38, 21	48	5.74	−42	4	−34	—
R calcarine gyrus	—	19	4.87	14	−60	16	—	L medial temporal gyrus	38	23	5.45	−24	12	−30	—
L inferior temporal gyrus	—	12	4.78	−56	−58	−24	—	R precentral gyrus	6	10	5.41	34	−14	64	—
								R superior frontal gyrus	8	29	5.41	18	22	46	—
								R superior orbital gyrus	47	16	5.14	18	18	−12	—
								L inferior frontal gyrus	46, 10	29	4.96	−42	40	20	—
								R medial cingulate cortex	32	12	4.93	14	10	40	—
								L calcarine gyrus	—	14	4.92	−10	−86	6	—
								R medial temporal gyrus	21	14	4.86	64	−26	−14	—
								L putamen	—	11	4.58	−30	−10	10	—
								L inferior frontal gyrus	46	14	4.52	−42	32	22	—
								L parahippocampal gyrus	—	10	4.27	−14	−14	−28	—

*−TPD_gain_*								*TPD_gain_*							
*Location*	*BA*	*voxel*	*T-value*	*x*	*y*	*z*	*P*_FWE_^∗^	*Location*	*BA*	*voxel*	*T-value*	*x*	*y*	*z*	*P*_FWE_^∗^
R cerebellum (IV, V)	—	21	7.01	18	−50	−22	—	L medial frontal gyrus	11	13	5.84	−44	52	−14	—
R superior medial gyrus	—	24	6.92	10	50	40	—	L medial frontal gyrus	—	13	5.74	−42	12	50	—
L medial occipital gyrus	19, 39	21	5.05	−46	−84	20	—	R hippocampus	—	12	5.27	32	−18	−12	0.040
R supramarginal gyrus	—	19	4.73	42	−36	36	—	L precuneus	7	26	5.09	−6	−54	48	—
L fusiform gyrus	19	15	4.72	−32	−48	−10	—	L postcentral gyrus	4	10	4.96	−62	−12	40	—
L medial temporal gyrus	22	15	4.13	−58	−32	4	—								

^∗^Small volume corrected on cluster level. BA: Brodmann area; L: left; R: right.
